# Increased cannabis intake during the COVID-19 pandemic is associated with worsening of depression symptoms in people with PTSD

**DOI:** 10.1186/s12888-022-04185-7

**Published:** 2022-08-17

**Authors:** A. Murkar, T. Kendzerska, J. Shlik, L. Quilty, M. Saad, R. Robillard

**Affiliations:** 1 University of Ottawa Institute of Mental Health Research at The Royal, Sleep Research Unit, 1145 Carling Ave, ON K1Z 7K4 Ottawa, Canada; 2 The Ottawa Hospital Research Institute, Ottawa, ON Canada; 3 The Royal Ottawa Mental Health Centre, Ottawa, ON Canada; 4 Centre for Addiction and Mental Health, Toronto, ON Canada; 5 Department of Psychiatry, University of Toronto, Toronto, ON Canada; 6 University of Ottawa School of Psychology, ON Ottawa, Canada

**Keywords:** COVID-19, Cannabis, PTSD, Stress, Anxiety, Depression

## Abstract

**Background:**

Some evidence suggests substance use affects clinical outcomes in people with posttraumatic stress disorder (PTSD). However, more work is required to examine links between mental health and cannabis use in PTSD during exposure to external stressors such as the COVID-19 pandemic. This study assessed mental health factors in individuals with self-reported PTSD to: (a) determine whether stress, anxiety, and depression symptoms were associated with changes in cannabis consumption across the pandemic, and (b) to contrast the degree to which clinically significant perceived symptom worsening was associated with changes in cannabis intake.

**Method:**

Data were obtained as part of a larger web-based population survey from April 3rd to June 24th 2020 (i.e., first wave of the pandemic in Canada). Participants (*N* = 462) with self-reported PTSD completed questionnaires to assess mental health symptoms and answered questions pertaining to their cannabis intake. Participants were categorized according to whether they were using cannabis or not, and if using, whether their use frequency increased, decreased, or remained unchanged during the pandemic.

**Results:**

Findings indicated an overall perceived worsening of stress, anxiety, and depression symptoms across all groups. A higher-than-expected proportion of individuals who increased their cannabis consumption reached threshold for minimal clinically important worsening of depression, X^2^(3) = 10.795, *p* = 0.013 (Cramer’s *V* = 0.166).

**Conclusion:**

Overall, those who increased cannabis use during the pandemic were more prone to undergo meaningful perceived worsening of depression symptoms. Prospective investigations will be critical next steps to determine the directionality of the relationship between cannabis and depressive symptoms.

## Background

Posttraumatic stress disorder (PTSD) is a psychiatric disorder that can arise following exposure to actual or threatened death, serious injury, or sexual violence. PTSD is notably characterized by intrusion symptoms (e.g., flashbacks, recurring nightmares, etc.), avoidant behaviors, negative alterations in cognition and mood, and alterations in arousal and reactivity [1]. PTSD is also highly comorbid with anxious and depressive disorders [2]. The COVID-19 pandemic has widely exacerbated stress, anxious, and depressive symptoms, and there are indications that people with PTSD were particularly affected by this global external stressor [3–6]. Of note, PTSD is a known risk factor for increased substance use [7–9]. Cannabinoids in particular have relevancy to PTSD both as therapeutic products [10–13] as well as drugs of abuse [7, 9]. Since the beginning of the COVID-19 pandemic, evidence has mounted suggesting that many factors such as stress about world events, long periods of confinement, and changes in sleep patterns have contributed to increased stress-related mental health problems [14–22], a phenomenon that may influence patterns of substance use. While some work has examined changes in substance use behaviours during the pandemic [23], little is yet known about the mental health factors associated with changes in cannabis use patterns among individuals with PTSD during this period.

Mental health factors have long been known to affect substance use. For instance, past research has identified that exposure to a traumatic event is related to the initiation of cannabis consumption [24]. PTSD-related mental health issues have also been highlighted as possible risk factors for substance use disorders [25–27]. Recent work has shown that among Canadian and Australian samples, combined usage of substances including alcohol and cannabis increased during the COVID-19 pandemic [28, 29] – and the perceived worsening of PTSD symptoms during the pandemic was associated with these increases [29]. Among Canadians, approximately one third of substance-using individuals also reported an increase in alcohol and cannabis intake during the pandemic [30]. Cannabis became widely available to Canadians in 2018 following the introduction of the *Cannabis Act*, which legalized the possession, purchase, sharing, and sale of cannabis for individuals over 18 years of age [31]. Thus, since that time point, cannabis has been sharing a similar availability and legal status to alcohol in Canada. There is a need to better understand the mental health correlates of changes in substance use in people with PTSD during this unique period where cannabis legislation was quickly followed by a major global stressor.

There is some evidence to suggest that psychiatric comorbidities can affect cannabis consumption in people with PTSD. Recent evidence has indicated that both depressive and anxious symptoms are associated with greater odds of cannabis use in people with PTSD [32]. Perceived stress is similarly associated with increased cannabis intake [33, 34], and the relationship between perceived stress and cannabis use is mediated by depression and anxiety in people with PTSD [35]. Additionally, stress, anxiety, and depression are cited as some of the most common reasons for cannabis use among PTSD-prevalent groups such as veterans [36]. There is also a physiological basis to suspect that cannabinoids may directly modulate brain processes contributing to PTSD symptomology as well as comorbid stress, anxiety and depression symptoms. Individuals with PTSD exhibit reduced availability of the endocannabinoid anandamide and a consequent upregulation of its receptor (CB1) [37], which plays a key role in the mediation of stress and fear responses at relevant brain sites [10, 38–43]. The endocannabinoid system is also believed to be implicated in stress [44, 45], anxiety [46, 47], and depressive symptoms [48, 49].

Due to its prevalent use either under medical authorization or as self-medication for PTSD, we speculated that cannabis use might change with increases in stress, anxiety, and depression symptoms co-occurring with PTSD during a global stressor like the COVID-19 pandemic. The present study aimed to assess mental health factors in a sample of individuals with self-reported current diagnoses of PTSD in order to determine whether changes in the severity of stress, anxiety, and depression symptoms during the pandemic relative to pre-pandemic estimates were associated with changes in cannabis consumption. We addressed this objective first by comparing symptoms of stress, anxiety, and depression among groups of individuals with PTSD who reported different cannabis intake behaviours across the pandemic (those who increased their intake, those who did not change their intake, those who decreased their intake, and those who did not use cannabis in the month prior to the pandemic or during the pandemic). Next, we compared the proportions of those whose stress, anxiety, and depression symptoms underwent *minimal clinically important differences* (MCID, a framework for characterizing the minimum level of symptom change that would be considered meaningful and that would mandate a change in illness management [50] between the cannabis groups).

## Methods

### Data collection

The data for this study were obtained as part of a larger web-based population survey on the psychological, social, and economic impacts of the COVID-19 pandemic that was circulated via websites, social media, and multiple organizations and hospitals across Canada (please see ClinicalTrials.gov: NCT04369690 and [21]). The data included in this report contains survey entries from April 3rd until June 24th 2020. The survey was available in both official Canadian languages (English and French) and included custom-built questions regarding the COVID-19 pandemic (please see a copy of these items in previously published data supplement [21]), as well as validated questionnaires regarding mental health. It was developed in accordance with the Checklist for Reporting Results of Internet E-Surveys (CHERRIES; [51]). Data collected for the study was collected in an anonymous manner. Retrospective questions were used to estimate stress, anxiety and depression symptoms, as well as cannabis use frequency across two time-referents: in the last month before the beginning of the COVID-19 pandemic (the pandemic declaration by the World Health Organization occurred on 11 March 2020), and in the 7 days prior to filling out the survey.

### Sample

The main inclusion criterion for the current sample was a self-reported current diagnosis of PTSD (i.e. selecting PTSD amongst a list of multiple mental disorders when answering the question: ‘Have you ever had a formal diagnosis of (Please select all that apply)’). 466 participants with self-reported current PTSD were identified from the overall sample of 6,981 participants (6.7%) who filled out the survey during that period. Four participants with self-reported current PTSD were excluded from the analysis as they met the following exclusion criteria: younger than 18 years of age or diagnosis of a psychotic disorder. The final sample consisted of 462 participants.

The sample of respondents was divided in four cannabis groups based on changes in cannabis use patterns from pre-pandemic to during the pandemic. Change scores were calculated by subtracting estimated pre-pandemic cannabis consumption frequencies from the frequencies reported during the pandemic. Individuals with a negative change score (i.e., a decrease in weekly cannabis consumption frequency from pre-pandemic to during the pandemic) were included in the decreased use group. Those with a positive change score (i.e., an increase in weekly cannabis consumption frequency) were included in the increased cannabis use group. Those with a change score of 0 were included in the no-change group. A cannabis non-user group was also formed to include the respondents who reported not having used cannabis at any of the sampled time referents.

### Questionnaires

Three validated questionnaires were used to assess symptoms of stress, anxiety, and depression, all of which have previously been utilized with people with PTSD [52–55]. The 10-item version of the Perceived Stress Scale (PSS10; [56]) was used to assess stress symptoms [57]. Participants answered questions on a five-point Likert scale, with total scores ranging from 0 to 40, where higher scores indicate worse perceived stress. The Cronbach α and test-retest of the PSS10 were both reported as greater than 0.70 [58]. The Generalized Anxiety Disorder 7 (GAD-7; [59]), a 7-item questionnaire, was used to assess the severity of symptoms of anxiety. Scores range from 0 to 21, with a higher score indicating a more severe anxiety. Internal consistency was found to be excellent (Cronbach α = 0.92) and test-retest reliability was good (intraclass correlation = 0.83). The GAD-7 was also found to have good sensitivity (89%) and specificity (82%) [59]. The QIDS-SR16 [60] was used to assess depression symptoms. The QIDS-SR16 is a questionnaire assessing the nine symptom domains of depression used in the DSM-IV. It contains 16 items for which respondents are asked to rate the severity of symptoms. Scores range from 1 to 27, with higher scores indicating more severe depression symptoms [61]. Based on a meta-analysis, the QIDS-SR16 was found to be unidimensional and to have an internal consistency (Cronbach’s α) ranging from 0.69 to 0.89 [62].

Regarding cannabis consumption, participants were asked the following questions: “How frequently were you taking cannabis products: Total number of times in the past 7 days? Number of times per week in the last month before the pandemic?”.

### Statistical analyses

Mixed ANCOVAs with cannabis groups (increased use, no change, decreased use, and non-users) as a between-subjects factor and time referent (before the pandemic and during the pandemic) as a repeated-measures factor were applied on PSS10, GAD-7 and QIDS-SR16 total scores. All ANCOVAs were adjusted for relevant covariates: age, occupation, and current diagnosis of addiction-related/substance use disorders. Levene’s test and Box’s test were used to ensure that equality of error variances and equality of covariance matrices were not violated.

The proportion of individuals whose perceived symptom worsening was above the threshold for MCID was compared across the cannabis groups using chi-squared tests which were subsequently broken down using Bonferroni-corrected z-tests according to the procedure described by Sharpe [63]. Cramer’s V was used as an effect size for these analyses [64]. For determining MCID, the thresholds for all three questionnaire assessments were based on previously established norms. For the PSS10, a 28.0% increase was identified as MCID [65]. For the GAD-7, an increase of four points was identified as MCID [66]. For the QIDS-SR16, an increase of 28.5% was identified as MCID [67].

## Results

### Sample characteristics

The descriptive data across the cannabis groups are reported in Table 1. The sample ranged between 18 and 78 years of age (mean ± SD = 48.02 ± 13.89), and was mostly composed of white (83.15%), female (71.3%) individuals. Sex and ethnicity distributions did not differ significantly between the cannabis groups. There was a significant difference between the cannabis groups in terms of age, F(3,458) = 5.602, *p =* 0.001 and occupation, X^2^(9)= 19.589, *p* = 0.021; those who did not change their cannabis use across the time referents (*p* < 0.05) and non-users (*p* < 0.05) were significantly older than those who decreased their use and counted a higher proportion of retired individuals compared to all other cannabis groups. In addition, there was a significant difference between the groups for current diagnosis of addiction-related/substance use disorders, X^2^(3)=10.197, *p* = 0.017. A significantly higher proportion of individuals who increased their cannabis use during the first wave of the pandemic had a self-reported diagnosis of a substance use disorder compared to non-users (*p* < 0.05).

**Table 1:** Demographic data for each of the four cannabis use groups

	Increased (*n* = 54)	No Change (*n* = 66)	Decreased (*n* = 40)	Non-cannabis (*n* = 303)	Overall (*n* = 462)	*p *(*η*^*2*^*p*/Cramer’s V)
Mean age, years (*SD*)	43.1 (*15.1*)	48.7 (*11.7*)	42.1 (*13.7*)	49.3 (*13.8*)	48.1 (*13.9*)	**0.001(*0.04*)
Sex, *n* male	14 (26%)	23 (35%)	15 (38%)	80 (26%)	132 (29%)	0.308 (0.09)
Income (> $100k/year)	36 (69%)	38 (58%)	27 (69%)	189 (64%)	290 (64%)	0.518 (0.07)
% White	45 (83%)	56 (86%)	33 (85%)	246 (82%)	381 (82%)	0.960 (0.08)
Education						0.094 (0.11)
University	25 (46%)	24 (36%)	13 (33%)	139 (46%)	201 (43%)	0.232(0.10)
College	15 (28%)	30 (45%)	12 (30%)	98 (32%)	155 (33%)	0.145(0.11)
Highschool or less	14 (26%)	12 (18%)	15 (38%)	65 (22%)	106 (23%)	0.100(0.12)
Occupation						*0.021 (0.12)
Employed	17 (32%)	25 (38%)	14 (36%)	120 (40%)	176 (38%)	0.722 (0.05)
Retired	8 (15%)	20 (30%)	7 (18%)	87 (29%)	122 (27%)	0.092 (0.12)
Student	6 (11%)	1 (2%)	5 (13%)	12 (4%)	24 (5%)	*0.011 (0.16)
Unemployed	22 (42%)	20 (30%)	13 (33%)	81 (27%)	136 (30%)	0.183 (0.10)
Psychiatric comorbidities					0.207 (0.10)	
Mood disorders	39 (72%)	39 (59%)	32 (80%)	207 (69%)	317 (69%)	0.138 (0.11)
Anxiety disorders	34 (63%)	40 (61%)	28 (70%)	166 (55%)	269 (58%)	0.237 (0.10)
Obsessive compulsive disorder	5 (9%)	6 (9%)	6 (15%)	26 (9%)	43 (9%)	0.634 (0.06)
Substance-related/ addictive disorders	9 (17%)	2 (3%)	4 (10%)	18 (6%)	33 (7%)	*0.017 (0.17)
Eating disorders	6 (11%)	2 (3%)	3 (8%)	20 (7%)	31 (7%)	0.370 (0.08)
Medications						0.722 (0.05)
Antianxiety	17 (37%)	18 (30%)	17 (45%)	99 (37%)	151 (36%)	0.492 (0.08)
Antidepressant	31 (67%)	36 (59%)	20 (53%)	159 (59%)	246 (59%)	0.579 (0.07)
Antihypertensive	5 (11%)	9 (15%)	5 (13%)	48 (18%)	67 (16%)	0.620 (0.07)
Respiratory therapy	12 (22%)	17 (26%)	3 (8%)	56 (18%)	88 (19%)	0.121 (0.11)

*Note.* * = *p* < 0.05; ** = *p* < 0.01

### Perceived stress

**Fig. 1:**
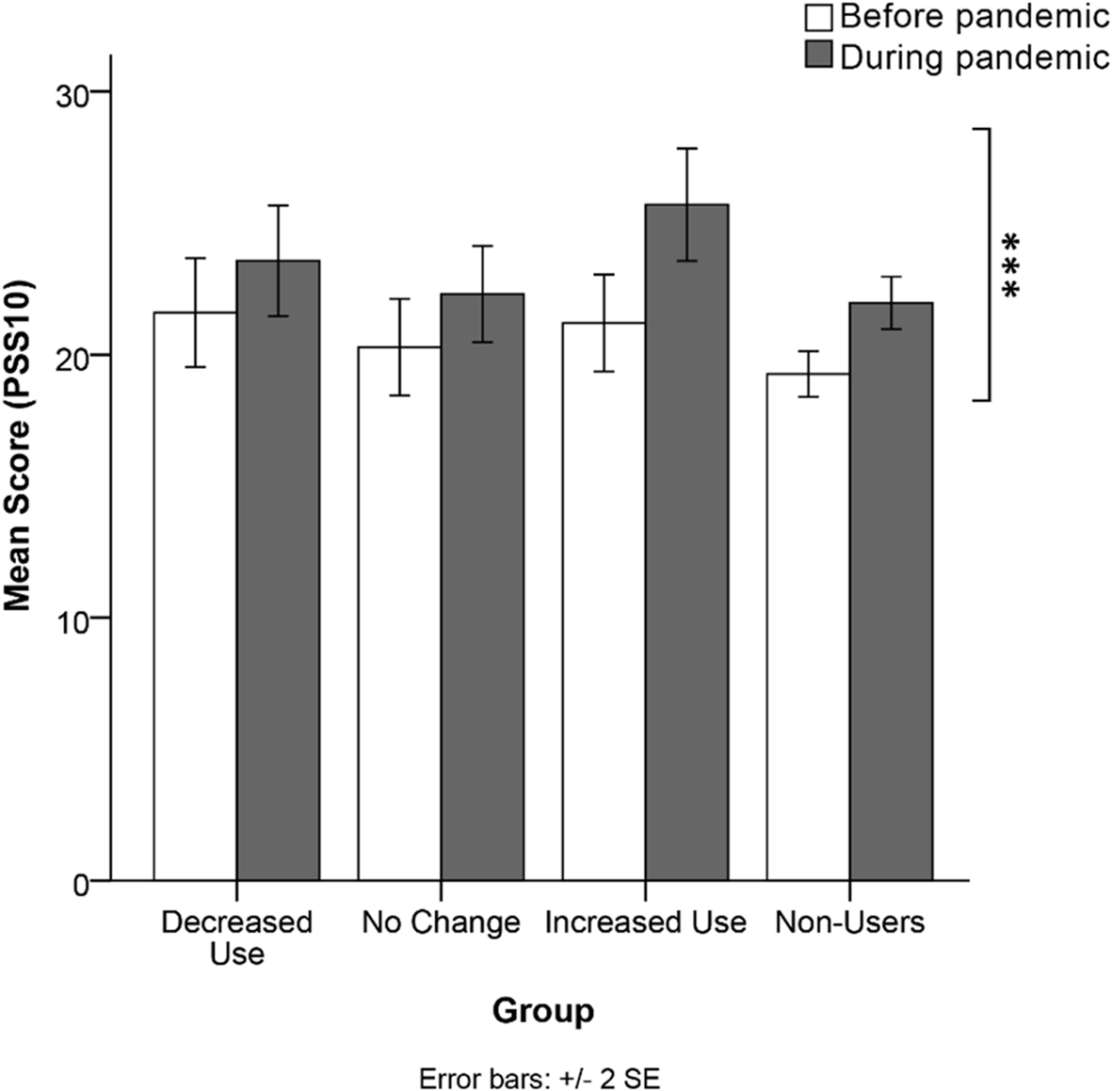
Comparison of  perceived mean stress symptoms scores on the PSS10 across the cannabis use groups at both time referents (before and during the pandemic). A significant main effect of time referent was observed on PSS10 scores, F(1,411) = 12.948, *p* < 0.001 with a small effect size (η^2^*p* = 0.031). Note.***p < 0.001

PSS10 scores prior to and during the pandemic in each cannabis group are depicted in Fig. 1. There was no significant interaction between time referent and cannabis group for the PSS10, F(3,411) = 0.978, *p* = 0.403, nor significant main effect cannabis group on perceived stress scores, F(3,411) = 0.778, *p* = 0.507. A significant main effect of time referent was observed on PSS10 scores, F(1,411) = 12.948, *p <* 0.001 with a small effect size (η^2^*p* = 0.031). Overall, there was a significant increase in PSS10 scores from before the pandemic (M = 20.93, SE = 0.63) to during the pandemic (M = 24.31, SE = 0.70).

### Anxiety symptoms

**Fig. 2:**
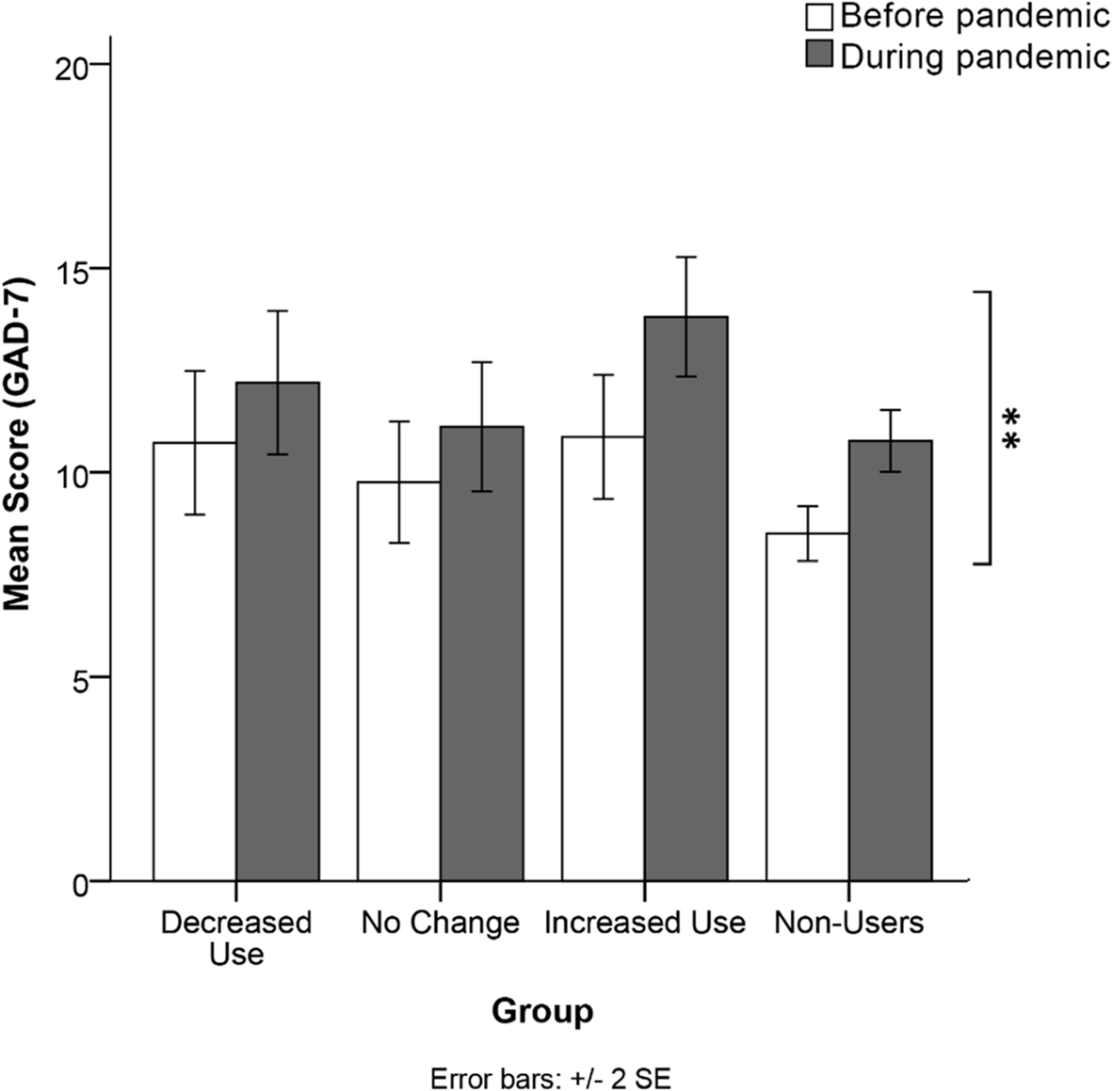
Comparison of mean anxiety symptoms scores on the GAD-7 across the cannabis use groups at both time referents (before and during the pandemic). A significant main effect of time referent showed that anxiety levels increased during the pandemic relative to pre-pandemic estimates in all groups. Note. **p = 0.002

Anxiety symptoms severity as reflected by GAD-7 scores across the time referents for each cannabis groups are depicted in Fig. 2. There was no significant time referent by cannabis group interaction for the GAD-7, F(3,409) = 0.805, p = 0.491, nor significant main effect cannabis group on GAD-7 scores, F(3,409) = 2.036, *p* = 0.108. A significant main effect of time referent on anxiety scores on the GAD-7, F(1,409) = 9.989, *p* = 0.002 with a small effect size (η2p = 0.024) revealed a significant increase in anxiety scores from before the pandemic (M = 10.149, SE = 0.51) to during the pandemic (M = 12.24, SE = 0.55).

### Depression symptoms

**Fig. 3:**
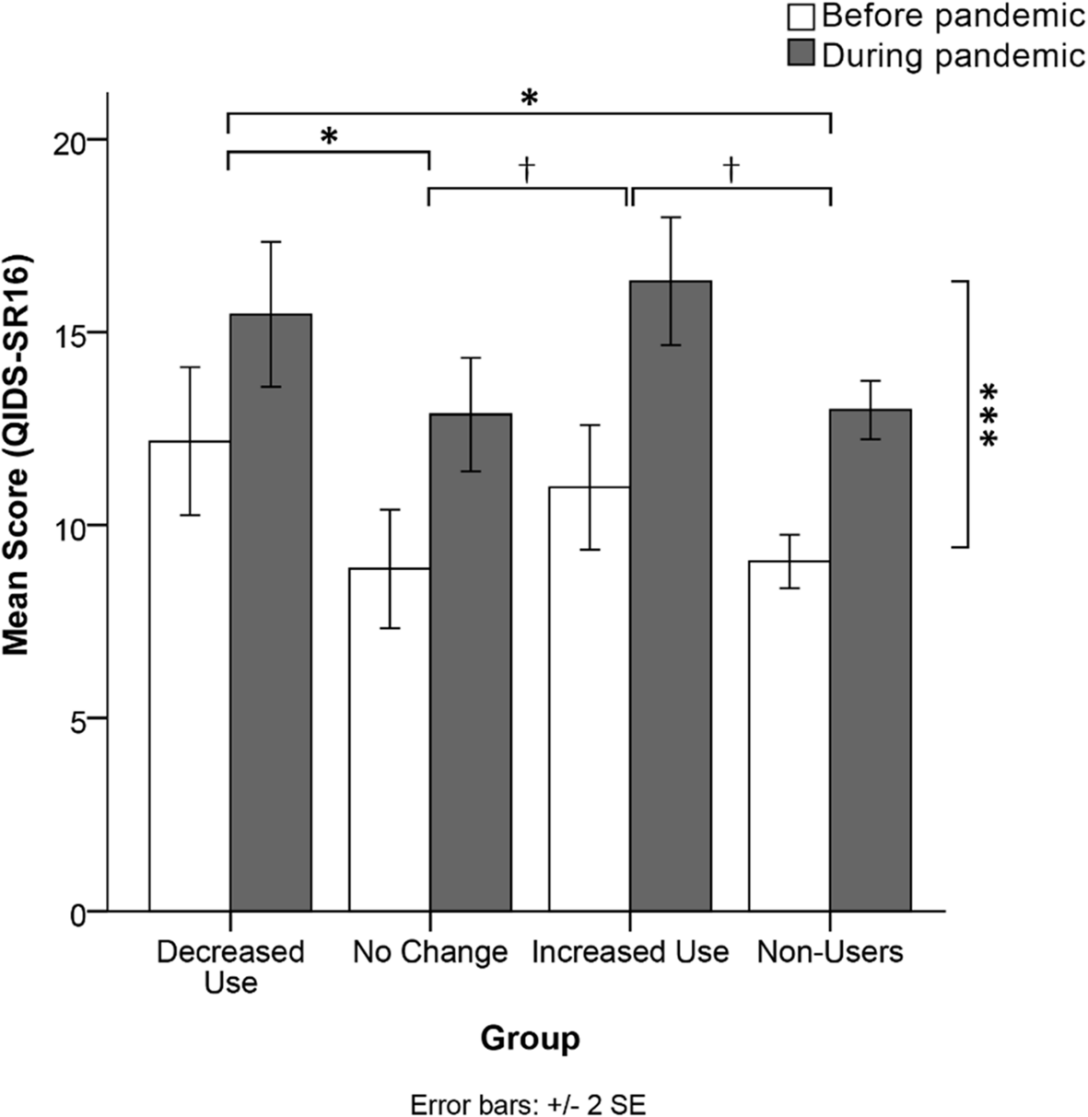
Comparison of mean depressive symptoms scores on the QIDS-SR16 across the cannabis use groups at both time referents (before and during the pandemic). A significant main effect of time referent showed that anxiety levels increased during the pandemic relative to pre-pandemic estimates in all groups. A significant main effect of cannabis group showed that those who decreased their cannabis use had more severe depression symptoms than those who did not change their use and those who did not use. There was also a non-significant trend (†) for those who increased their cannabis use toward having more severe depression symptoms compared to those who did not change their use during the pandemic and non-users. Note.*p < 0.05, ***p < 0.001, †non-significant trends (*p < *0.06)

Figure 3 shows depression symptoms severity as reflected by QIDS-SR16 scores prior to and during the pandemic for each cannabis group. There was no significant time referent by cannabis group interaction effect for QIDS-SR16 scores, F(3,411) = 0.805, *p* = 0.491. There was a significant cannabis group difference for QIDS-SR16 scores, F(3,383) = 2.903 *p* = 0.035 with a small effect size (η^2^p = 0.022). Post-hoc analyses revealed that those who decreased their cannabis use (M = 13.106, SE = 0.741) had significantly more severe depression symptoms compared to those who did not change their use during the pandemic (M = 10.974, SE = 0.602), *p* = 0.017, and those who did not use cannabis (M = 11.314, SE = 0.272), *p* = 0.016. Additionally, there was a non-significant trend for those who increased their cannabis use (M = 12.681, SE = 0.650) toward having more severe depression symptoms compared to those who did not change their use during the pandemic (*p* = 0.055) and non-users (*p* = 0.056). No other between-groups differences or trends emerged from the analysis.

Our findings also revealed a significant main effect of time referent on depression scores on the QIDS-SR16, F(1,383) = 21.590, *p <* 0.001 with a small effect size (η^2^p = 0.053). Overall, there was a significant increase in depression scores from before the pandemic (M = 10.85, SE = 0.54) to during the pandemic (M = 15.17, SE = 0.56).

### Clinically important worsening

**Fig. 4:**
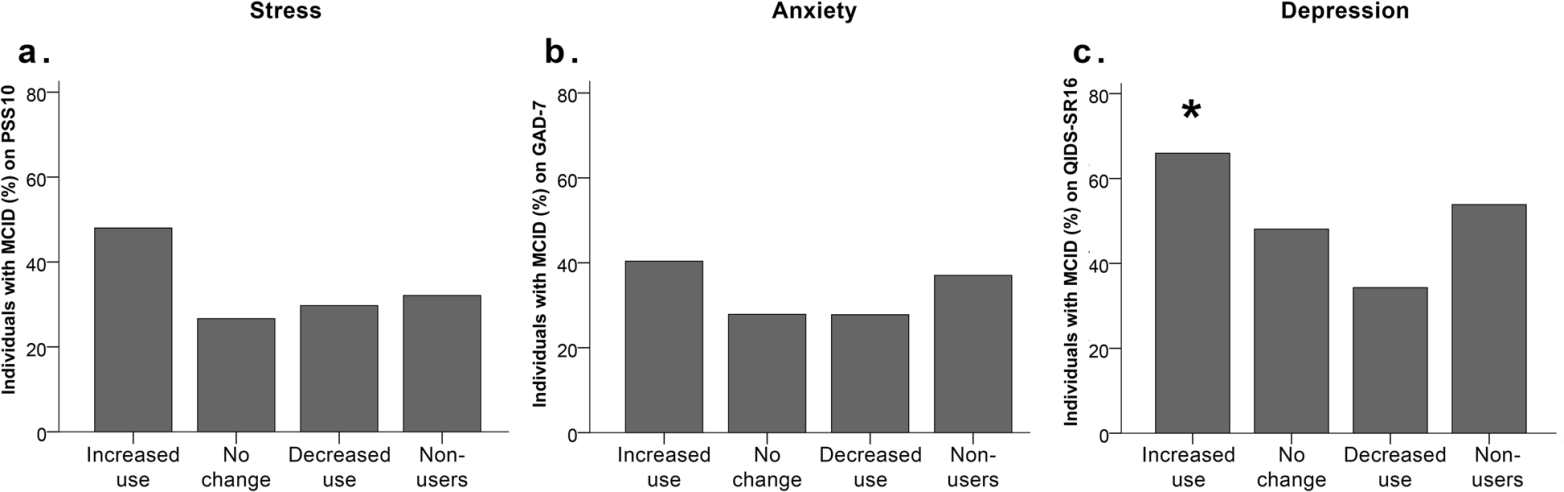
Proportions of individuals with MCID across the cannabis use pattern groups on (**A**) PSS10, (**B**) GAD-7, and (**C**) QIDS-SR16. Only the QIDS-SR16 differed significantly across groups: a higher-than-expected proportion of individuals who increased their cannabis consumption during the pandemic reached the MCID threshold for depression worsening. Note.* = *p *< 0.05

There was no significant difference across the cannabis groups in the proportions of individuals with MCID reflecting perceived stress or anxiety symptoms worsening based on retrospective estimates, PSS10: Chi-squared (3) = 6.886, *p* = 0.076 (Fig. 4a), GAD-7; Chi-squared (3) = 4.716, *p* = 0.194 (Fig. 4b). However, there were significant cannabis groups differences in the proportions of those with clinically significant worsening of depression symptoms based on retrospective estimates, QIDS-SR16: Chi-squared (3) = 10.795, *p* = 0.013 with a small effect size (Cramer’s *V* = 0.166; Fig. 4c). Specifically, a higher-than-expected proportion of individuals who increased their cannabis consumption during the pandemic reached the MCID threshold for depression worsening (72%), *p* < 0.05 (see Fig. 4c). No such difference was observed for those who did not change their cannabis use (46%), those who decreased their use, (40%), and non-users (50%).

## Discussion

Intra-individual changes from before the pandemic to during the pandemic are of significant interest in terms of understanding the dynamic relationship between mental health factors and cannabis consumption in the context of a global crisis. Here, we have utilized a retrospective approach with data sampled in the early stages of the first wave of the pandemic. Overall, our findings revealed a significant increase in stress, anxiety, and depression symptom severity among all cannabis groups during the first wave of the pandemic relative to self-reported retrospective pre-pandemic estimates. Only overall depression scores differed significantly among the cannabis groups. Specifically, we found that compared to non-users and those who maintained a stable frequency of cannabis use during the pandemic, those who decreased or increased (trend) their cannabis use experienced higher depression symptoms both before and during the pandemic (i.e. no significant interaction with pandemic-related changes in depression). Those who used cannabis but did not change their frequency of use during the pandemic maintained the lowest QIDS-SR16 scores across this period. Our analysis also revealed that clinically important worsening of depression (based on retrospective estimates) was related to increased cannabis use in individuals with a self-reported current diagnosis of PTSD; no such association was observed for stress or anxiety.

Our findings indicated that 72% of those who increased their cannabis use during the pandemic experienced clinically important worsening of depression symptoms (based on retrospective estimates), compared with 40–50% for all other groups. Since we did not observe any significant interaction between cannabis use and retrospectively estimated pandemic-related changes in continuous variables reflecting symptom severity in our first analyses, the relationship between depressive symptoms worsening and increased cannabis use during the pandemic may have been more pronounced in cases of prominent and clinically significant symptom worsening rather than in cases of sub-clinical symptom changes. Lastly, we did not observe any significant differences in the proportions of those who experienced clinically important worsening of stress or anxiety among the cannabis use groups. Thus, it appears that depression symptoms alone were chiefly associated with changes in cannabis use patterns in the face of this external stressor.

Our findings with regards to depression may be consistent with other research on cannabis-use patterns during the pandemic. Recent evidence has identified self-isolation and coping with depression as primary drivers behind cannabis buying practices during the pandemic in a non-PTSD sample [68]. Our work would therefore appear to support those results for individuals with PTSD who experienced clinically meaningful worsening in depression symptoms (based on retrospective estimates), suggesting that depression symptoms are an important factor associated with cannabis intake in PTSD during a global crisis (an observation that is particularly relevant given the high comorbidity of PTSD and depression [69, 70]).

Our findings regarding stress may also be somewhat consistent with past research. Previous work has demonstrated for example that cannabis use may be associated with a blunted stress response [71]. Regarding anxiety, however, the evidence is less clear-cut. In the short-term, high doses of tetrahydrocannabinol (THC, the major psychoactive constituent of cannabis) are anxiogenic [72] while low doses are anxiolytic [42, 73, 74]. Meta-analyses indicate that, in the long-term, there is a weak but positive relationship between cannabis use and anxiety [75] (although it is unclear whether anxiety is a main motivating factor for cannabis use in PTSD specifically [76]). Some work has also suggested that the largest increases in medical cannabis intake during the pandemic were observed in participants with pre-existing anxiety [77]. We did not observe this in our sample, however, as there were no significant differences between our cannabis groups in terms of either GAD-7 scores or comorbid anxiety disorders. Interestingly, multiple studies have indicated that the relationship between cannabis and anxiety may also be specific to social anxiety disorder [78, 79]. While our questionnaire measures did not specifically isolate social anxiety symptoms, we did not observe high levels of self-reported current diagnoses of social anxiety disorder in this sample among any of the cannabis-using groups.

It remains unclear whether cannabis use may increase as a result of therapeutic use/self-medication or whether it instead may be a driving factor behind symptoms changes. Given the evidence of the association between the pandemic and worsening of mental health [80–82], it is possible that cannabis consumption was increased as a coping strategy in response to mental health challenges imposed by this global external stressor. Previous evidence has highlighted coping as a motive of cannabis use [76, 83–85], and recent work has more specifically indicated that cannabis users with greater difficulty tolerating sad mood states use cannabis more frequently [85]. Thus, one possibility in the current study is that, among those with lower resiliency to sad mood states, worsening of depressive symptoms may have motivated increased cannabis use as a coping strategy during the onset of the pandemic. However, research also points to cannabis as a risk factor for depressive symptoms [86]. The relationship between cannabis use and depression may thus be bidirectional. It is also worth noting that comorbid substance use could also have played a role in these effects (since other studies have reported increased use of both alcohol and cannabis among substance-using individuals during the pandemic [30]).

Interestingly, those who reported not changing their levels of cannabis use during the onset of the pandemic also reported the lowest overall depression symptoms. Indeed, some positive effects of cannabinoids have been shown for PTSD (e.g., the reduction of PTSD-related nightmares by Nabilone [12]). Since PTSD may be characterized by reduced levels of the endocannabinoids anandamide and a compensatory upregulation of central CB1 receptors [87], it is possible that supplementation with stable consumption of THC (which, like anandamide, acts as a CB1 partial agonist [42]) could re-normalize the central endocannabinoids system. Conversely, acute changes in cannabis consumption may alter this balance. However, more work is needed in this area since there is a dearth of information on the effects of THC on CB1 receptor expression in humans.

The present study has several limitations. Firstly, the information about cannabis use was limited to weekly frequency of use, and no information was available about other important factors such as dosage, the ratio of CBD/THC concentrations, or medical versus recreational use which are speculated to play an important role in responses to cannabis use [88]. This limitation is particularly important given the known differences in the effects of dosage and CBD versus THC concentrations on behaviour and emotion as well as recent concerns raised about the lack of adequate standardization of cannabinoid-based products [10, 89, 90]. Secondly, this study relied on self-reported diagnosis without clinician-based confirmation, and it was also based on retrospective assessment of pre-pandemic mental health and substance use. Because of the spontaneous nature of the pandemic, obtaining a prospective sample from before the pandemic was not possible. Our results may therefore be affected by recall bias. In addition, the nature of self-report measures is such that participants could omit or misreport information pertaining to their diagnoses or cannabis intake (which is a concern in all studies relying on self-report measures). Thirdly, the generalizability of this research is also somewhat restricted by the high proportion of females. Mian et al. [91] recently reported that females are significantly more likely to participate in survey-based cannabis research than males. Although the current data is issued from a larger survey which was not focused on cannabis, it is possible that volunteer bias influenced participant’s responses to this portion of the survey. For future research, it may be important to achieve more representative samples by oversampling groups which are more difficult to reach. This sample was also biased towards middle age white individuals, many with high total family income levels. Fourthly, while our study had adequate sample sizes overall, a larger sample would have allowed for more in-depth characterization of the data across the groups (particularly with regards to comparing those with clinically meaningful worsening in mental health (based on retrospective estimates) versus those who did not in a between-groups manner). Fifthly, several statistical tests were conducted, increasing the risks of type I error, and the observational nature of the study precludes any inference of causality. Finally, while the data reported in this sample were restricted to individuals with self-reported PTSD, we cannot rule out the possibility that our findings could also be more generalized to other populations. Overall, our findings support and expand upon prior work regarding substance use in individuals with PTSD and during the COVID-19 pandemic.

## Conclusion

This work highlights the association between clinically significant worsening depressive symptoms and increased cannabis intake among individuals with PTSD during the early phases of the pandemic. This research adds to the knowledge of potential factors linked to increased substance use in people with PTSD following a major crisis. Future work should aim to prospectively determine whether depression or other comorbidities not addressed in this study are predictive of longitudinal changes in cannabis intake in people PTSD over the remaining course of the pandemic and, most importantly, whether this is accompanied by further worsening in mental health outcomes or whether cannabis may help stabilize certain types of psychiatric symptoms in the context of an external stressor.

## Data Availability

Data could be made available upon request and formal data sharing agreement. Requests should be submitted to Rebecca Robillard at Rebecca.robillard@uottawa.ca.

## Abbreviations

PTSD: Posttraumatic stress disorder
CB1: Cannabinoid receptor type 1
MCID: Minimal clinically important difference
CBD: Cannabidiol
THC: Delta-9-Tetrahydrocannabinol
